# Establishment of pelvic inflammatory disease model induced by vaginal injection of *Ureaplasma urealyticum* liquids combined with fatigue and hunger

**DOI:** 10.1590/1984-3143-AR2022-0106

**Published:** 2023-10-30

**Authors:** Pengfei Liu, Xiao Yu, Jinjin Wang, Li Wang, Yi Ding, Jinxing Liu

**Affiliations:** 1 First College of Clinical Medicine, Shandong University of Traditional Chinese Medicine, Jinan, China; 2 Department of Gynecology, Affiliated Hospital of Shandong University of Traditional Chinese Medicine, Jinan, China; 3 Department of Gynecology, The Second Affiliated Hospital of Shandong University of Traditional Chinese Medicine, Jinan, China

**Keywords:** pelvic inflammatory disease, microorganism, inflammatory cytokines, adhesion

## Abstract

Pelvic inflammatory disease (PID) is an inflammation of the upper genital tract. PID is the leading cause of some severe sequelae in the absence of timely and accurate diagnosis and treatment. An appropriate animal model is needed to explore the underlying mechanism of PID sequelae. This study introduced an animal model of PID by vaginal injection of liquid *Ureaplasma urealyticum* combined with fatigue and hunger (UVF). This study was designed to test the feasibility of a rat model. A rat model was established using UVF irradiation. Levels of some inflammatory cytokines in the serum and the homogenates of the fallopian tubes were measured by ELISA, RT-PCR, and flow cytometry and compared with another rat model of *Ureaplasma urealyticum* liquids injected into the two uterus horns during laparotomy. Inflammatory alterations and adhesions were observed after hematoxylin and eosin (H&E) staining and detected using the Blauer scoring system. The results showed that the combined UVF and rat model caused apparent obstruction, edema, and adhesion in the fallopian tubes and connective tissues. The rat model showed upregulated CD4, CD8, and CD4/CD8 in peripheral blood mononuclear cells (PBMCs) and significantly increased levels of IL-4, IL-6, IL-10, and IL-17. UVF also enhanced the expression of tumor necrosis factor (TNF)-α, transforming growth factor (TGF)-β, vascular endothelial growth factor (VEGF) β, and matrix metalloproteinase (MMP)-2 (*P*<0.05). The UVF rat model can induce inflammatory alterations in the fallopian tubes and connective tissues, and can be used as a model of PID.

## Introduction

Pelvic inflammatory disease (PID) is a common condition characterized by inflammation of the upper genital tract (Brunham et al., 2015). Typical symptoms include pelvic pain, edema of the fallopian tubes, and endometrial inflammation (Haggerty et al., 2016). Furthermore, PID is the leading cause of further severe sequelae such as infertility and ectopic pregnancy under improper or delayed diagnosis and treatment (Curry et al., 2019). Such sequelae influence women's regular work and lives and create a tremendous economic burden. Therefore, it is essential to explore the development of PID and prevent further sequelae. Moreover, an appropriate animal model is needed to evaluate the effects of treatment or further development of PID.

PID is caused by the ascending inflammation of certain bacteria in the vagina (Ravel et al., 2021). Animal models of PID have been established for *Chlamydia* genital infections (De Clercq et al., 2013; Ross et al., 2018). However, reports indicate that *Chlamydia*, *Clostridium sordellii*, and lipopolysaccharide (LPS) may influence the overall health of experimental animals, including systemic symptoms and even death (Sheldon et al., 2010; Vanrompay et al., 2006). In addition, PID caused by these factors usually induces acute inflammation (Oh et al., 2016). However, these models differ from the reality. This study aims to establish an animal model of PID and detect related indices such as inflammatory cytokines and adhesion, obstruction, and edema of the fallopian tubes and connective tissues. The current study will measure whether the rat model of the vaginal injection of liquid *Ureaplasma urealyticum* combined with fatigue and hunger can induce the inflammatory alterations. We hope that the model can match PID symptoms and help prevent their occurrence as the search for a potentially effective therapy continues.

## Methods

### Ethics statement

All procedures were performed under the International Guiding Principles for Animal Research, and adopted by the Experimental Animal Center, Shandong University of Traditional Chinese Medicine. All protocols were approved by the Ethics Committee on Animal Experiments at Shandong University of Traditional Chinese Medicine (2021-017).

### Approval for animal experiments

The study is reported in accordance with ARRIVE guidelines.

### Materials

*Ureaplasma urealyticum* strains were purchased from the Shandong Province Skin Disease Venereal Disease Prevention and Treatment Institute. First, fetal bovine serum was mixed with a nine-part Nutrient Broth (HB0108, QINGDAO,) to produce a broth containing 10% fetal bovine serum. The broth was filtered, sterilized, and maintained at a pH of 6.2. The strains were then returned to 37 °C from -70 °C using a warm water bath. The strains were then placed in the liquid broth and sealed after the inspection of their activities. Finally, the strains were purified by repeated addition into the broth at 37 °C and diluted to 10^3^ CCU (color change unit) /mL after remaining under sterile conditions at 37 °C for a period of 24 h.

### Intervention method

Twelve-week-old virgin female Wistar rats weighing 220±20 g were purchased from Shandong University Laboratory Animal Shelter (Shandong, China). The rats were housed in individual cages under controlled environmental conditions (22±2 °C, relative humidity 40-60%, 12-h dark/light cycles, food, and water *ad libitum*). All the rats were treated in parallel for daily manipulation. After one week of acclimatization, the rats were first randomly divided into rats with and without fatigue and hunger treatment for 12 days.

On the 12th day, the rats were subsequently divided into five groups randomly:

the rats after treatment with fatigue and hunger received a 0.5 mLvaginal injection of *Ureaplasma urealyticum* liquid three times (UVF) (n=30);
the rats after treatment with fatigue and hunger received no vaginal injection (F) (n=30);
0.1 mL *ureaplasma urealyticum* liquids were injected into the two uteri horns of rats without fatigue and hunger treatment during the operation (UP) (n=30).
0.5 mL of 0.9% normal saline vaginal injection into the two uteri horns of rats without fatigue and hunger treatment during the operation (UV) (n=30).

The rats without fatigue and hunger treatment didn’t receive operation (C) (n=30).

The volume of each injection was approximately 0.5 mL every day due to the weight of the animal, and was administered on days 12, 13, and 14.

Short-term hunger and fatigue intervention: The rats were not fed on odd days, but provided food on even days. Fodder was supplied at 1/10 of the average weight of the rats. The rats performed basic swimming exercises for one hour every day for two days before the intervention. The rats were then placed in a 120×80×100 cm^3^ container and allowed to swim at a depth of 60±2 cm and temperature of 34±2 °C. The rats swam at 9 am along with a heavy metal weight of 12 g in groups of ten for one hour every day. The rats actively swam initially and then became exhausted. The exercise ended when the rats sank in water for over 10 s. After resting for 3 min, rats resumed their activities.

Establishment of the PID model by vaginal injection: After intervention for 12 days, rats were fixed in the supine position on the operating table, and the vaginal opening was fully exposed. Bacterial liquid (0.5 mL) was gently and slowly placed into the vagina after lubrication with paraffin oil. After the bacterial liquid did not flow from the vagina, rats were returned to their cages. They were injected at fixed times on days 12, 13, and 14.

Establishment of the PID model by operation: The rats were placed, and the two horns of uterus was removed under anesthesia with 7% chloral hydrate (5 mL/kg, intraperitoneal injection). The uterine endometrium was scraped, and 0.1 mL bacterial liquid was injected into the two uteri and in the directions of the two fallopian tubes and ovaries. The incision was then sanitized daily.

### Adhesive evaluation

Rats were euthanized on day 25. Then the adhesion of the fallopian tubes and connective tissue in the pelvis based on Blauer scoring (Tan et al., 2006) and the evaluation was as follows: 0- clear boundaries of tissues without adhesions; 1- vague boundaries and adhesions easily stripped; 2- thickening and hard to strip adhesions; 3- thickening and inability to strip adhesions, even those involved in the peritoneum and intestinal cavity.

### Histology staining

The experiments were performed on fallopian tubes with hematoxylin and eosin (H&E) (Servicebio, Wuhan, China) to identify inflammation in model rats. Briefly, fallopian tubes were fixed in 4%polyformaldehyde (Servicebio, Wuhan, China) for 24 h. The tubes were then cut into 4-μm sections, and three sections were stained with H&E. Two experienced pathologists observed the sections under an optical microscope (Nikon, Tokyo, Japan).

### Flow cytometry

Blood collected from the rat's abdominal aorta was placed in a test tube containing anticoagulant and centrifuged by ficoll (Stemcell, NewYork, USA) gradient according to the manufacturer's instructions. The red cell lysis (Biyuntian, Shanghai, China) was then added, incubated, and centrifuged at 1500 rpm for 5 min to obtain PBMC (peripheral blood mononuclear cell). PBMC was resuspended and added APC anti-rat CD3, FITC anti-rat CD4, or PE anti-rat CD8 (Baiaosi, Beijing, China), and incubated in darkness for 30 min. After washing with PBS, the PBMC was diluted with 500 μL PBS. The expressions of CD3+CD4+ and CD3+CD8+ cells were then measured by computer manipulation (BDinflux, NewYork, USA).

### ELISA assay

Blood samples were taken from the abdominal aorta of rats using Vacutainer (Kangweishi, Shijiazhuang, China). The blood samples from rats in different groups were centrifuged at 3000 rpm for 15 min and the serum was stored at -80 ° C for further detection. The levels of IL-4, IL-6, IL-10, and IL-17 in the serum were determined using a commercial ELISA kit (Wohong, Shanghai, China) according to the manufacturer’s instructions. The optical density (OD) was measured at a wave length of 450 nm, and the concentration was calculated based on the standard curve. Each experiment was performed in triplicate.

### RNA extraction and real-time RT-PCR assay

Total RNA was extracted using TRIzol reagent (Takara, Kyoto, Japan) according to the manufacturer’s instructions. The amount of RNA isolated from the homogenates of the ovaries and fallopian tubes was measured spectrophotometrically at 260 nm. RNA (500 ng) was reverse transcribed into complementary DNA (cDNA) using a commercial RT-PCR kit (Takara, Kyoto, Japan) in a volume of 20 μL at 37 °C for 15 min and 85 °C for 5 s. Quantitative real-time PCR was performed on a LightCycler 2.0 instrument (Roche Applied Science, Mannheim, Germany) using a SYBR Green I Reagent Kit (Toyobo, Tsuruga, Japan). The primers for tumor necrosis factor (TNF)-α (Tang et al., 2017) were 5’-ATGGGCTCCCTCTCATCAGT-3’ (sense) and 5’ - GCTTGGTGGTTTGCTACGAC-3’ (antisense); the primers for transforming growth factor (TGF)-β (Iturra et al., 2018) were 5’- GACCGCAACAACGCAATCTA’-3’ (sense) and 5’ - CGTGTTGCTCCACAGTTGAC -3’ (antisense). The primers for vascular endothelial growth factor (VEGF)β (Tham et al., 2006) were 5’ - GATCCAGTACCCGAGCAGTCA -3’ (sense) and 5’ - TGGCTTCACAGCACTCTCCTT -3’ (antisense) and those for matrix metalloproteinase (MMP)-2 (Feng et al., 2019) were 5’ - TTTGGTCGATGGGAGCATGG -3’ (sense) and 5’ - AGTACTCGCCATCAGCGTTC -3’ (antisense). The primers used for β-actin (Zhang et al., 2012) were 5’ -CGTTGACATCCGTAAAGACC-3’ (sense) and 5’ -TAGAGCCACCAATCCACACA-3’ (antisense). The primers were bought from Shenggong (Shanghai, China). The PCR reaction for TNF-α, TGF-β, MMP-2, VEGFβ, and β-actin was performed as follows: initial denaturation at 95 °C for 10 s, followed by 45 cycles of 60 °C for 5 s, 72 °C for 10 s, and termination by a cooling step for 30 s at 65 °C. β-actin was used as an internal control. Melting curve analysis was used to confirm the amplification specificity. Quantification data were analyzed using the Light Cycler analysis software (version 4.0; Roche Applied Science, Mannheim, Germany). Relative expression was normalized to that of β-actin. All experiments were performed in triplicate.

### Statistical analysis

SPSS software (version 23.0) and GraphPad Prism software (version 9.0; GraphPad Software, Inc., La Jolla, CA, USA) were used for data analysis. The Shapiro-Wilk test and Levene test were used to test the normal distribution of the data and the chi-square test. Data conformed to normal distribution and met chi-square, and one-way analysis of variance (ANOVA) and Tukey's test were used for comparison between groups. If the variance was not equal, Dunnett's T3 test was used. If the data did not conform to a normal distribution, the Kruskal-Wallis test was used. The chi-square test was used for comparison between groups of count data. Differences were considered statistically significant at p<0.05.

## Results

### The UVF rat model led to histopathological alterations and adhesion

**Figure 1:**
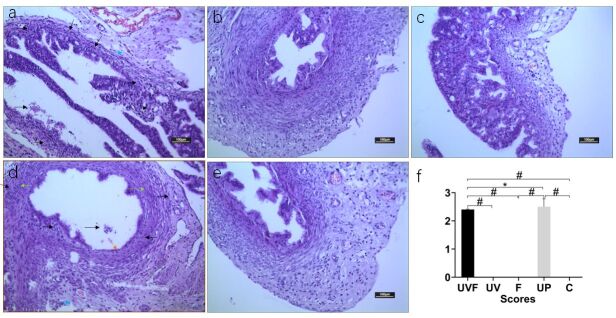
The representative histopathological alterations and adhesive evaluation. **a** displayed rat model of vaginal injection of *Ureaplasma urealyticum* combined with fatigue and hunger led to some chronic inflammatory alterations by H&E staining. The fallopian tube was dilated, edema was present (blue arrows), and there was an obstruction with more lymphocytes and neutrophil infiltration (black arrows). **b** H&E staining of the UV rats**. c** H&E staining results of rats in group F. **d** H&E staining results of UP rats. Epithelial cells are dropped (orange arrows). There was the significant proliferation of fibroblasts (green arrows) with lymphocytes and neutrophil infiltration (black arrows). **f** Adhesive scores of experimental rats in all groups during operation. There was clear boundary without obvious adhesions in the UV, F, and C groups. The adhesive scores were zero in the UV, F, and C groups. There were adhesions involving the pelvic and abdominal organs in the UVF and UP groups. Scale bars are 100μm. The UVF and UP groups had significantly higher scores than the UV, F, and C groups (#*P*<0.01). The adhesive scores in the UVF group were significantly lower than those in the UP group (*P<0.05). UVF, vaginal injection of *Ureaplasma urealyticum* liquid combined with fatigue and hunger; UV, rats with vaginal injection of *Ureaplasma urealyticum* liquids; UP, *Ureaplasma urealyticum* liquids were injected into the two uteri during the operation; F, rats under fatigue and hunger; C, control.

The results of H&E staining showed that vaginal injection of liquid *Ureaplasma urealyticum* combined with fatigue and hunger could cause some adhesions. The results of H&E staining showed that vaginal injection of liquid *Ureaplasma urealyticum* combined with fatigue and hunger could cause some chronic inflammatory alterations. As shown in Figure 1a, the fallopian tube was dilated, edema was present, and there was an obstruction with the lymphocytes and neutrophils infiltration. The epithelial cells were detached. Figure 1d also shows that the number of epithelial cells decreased. There was significant proliferation of fibroblasts with lymphocytes and neutrophils infiltration. During the operation of euthanized rats on day 25, we found there was clear boundary without obvious adhesions in the UV, F, and C groups. The adhesive scores were zero in the UV, F, and C groups. There were adhesions involving the pelvic and abdominal organs in the UVF and UP groups. The UVF and UP groups had significantly higher scores than the UV, F, and C groups (#*P*<0.01). The adhesive scores in the UVF group were significantly lower than those in the UP group (*P<0.05). The experimental adhesion’ scores of the experimental rats in all groups are shown in Figure 1f. The scores in groups UV, F, and C were zero (Figure 1b,c,e). The UVF and UP groups had significantly higher scores than the UV, F, and C groups (#*P*<0.01). In addition, the adhesive scores in the UVF group were significantly lower than those in the UP group (*P<0.05).

### *Ureaplasma urealyticum* liquid injection combined with fatigue and hunger impaired cellular immune balance

**Figure 2:**
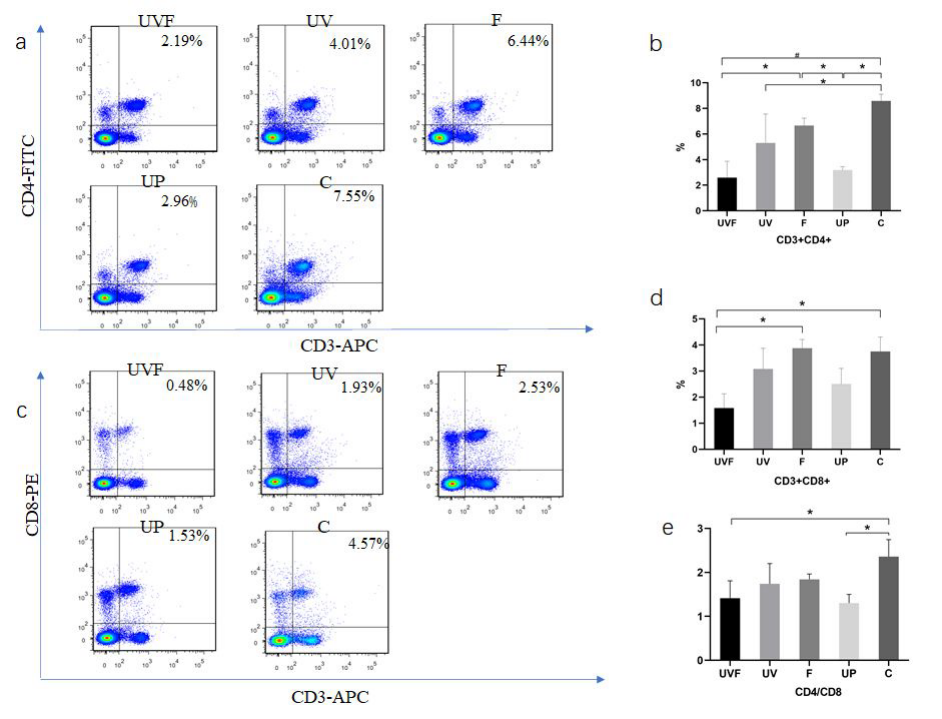
Bar graphs for cellular immune imbalance. a-e show the expressions of CD4, CD8, CD4/ CD8 cells by flow cytometry. a,b show the expression of CD4 indicated by CD3+CD4+ by flow cytometry according to group (UVF, UV, F, UP, and C). The x-axis indicates the expression of CD3+ indicated by APC, and the y-axis represents the levels of CD4+ indicated by FITC. **Figure 2a** suggests that *Ureaplasma urealyticum* liquid injection combined with fatigue and hunger attenuates the proportions of CD4+ indicated by CD3+CD4+ in PBMCs. The proportions of CD4+ in the UVF, UV, F, UP and C groups were 2.19%, 4.01%, 6.44%, 2.96%, and 7.55%, respectively. The proportions of CD8 indicated by CD3+CD8+ in the PBMCs of the UVF group diminished compared to those in the C group, as shown in **Figure 2d. Figure 2c** shows that the proportions of CD8+ cells were 0.48%, 1.93%, 2.53%, 1.53%, and 4.57% in the UVF, UV, F, UP and C groups, respectively. The proportions of CD4/ CD8 cells are shown in **Figure 2e.** UVF, vaginal injection of *Ureaplasma urealyticum* liquid combined with fatigue and hunger; UV, rats with vaginal injection of *Ureaplasma urealyticum* liquids; UP, *Ureaplasma urealyticum* liquids were injected into the two uteri during the operation; F, rats under fatigue and hunger; C, control.

Figure 2 a,b suggest that *Ureaplasma urealyticum* liquid injection combined with fatigue and hunger attenuates the proportions of CD4+ indicated by CD3+CD4+ in the PBMCs. As indicated, the proportions of CD4+ in group UVF are 2.19%, while ones in group C are 7.55%. Only *ureaplasma urealyticum* liquid injection suppressed the expression of CD4 in group UV and the proportions are 4.01%. The proportions of CD4+ in group F are 6.44% and ones in group UP are 2.96%. Similarly, the proportions of CD8 indicated by CD3+CD8+ in the PBMCs of group UVF diminished dramatically compared to those in C, as shown in Figure 2c,d. The proportions of CD8+ were 0.48% and 4.57% in group UVF and group C, respectively. The proportions of CD8+ were mitigated to 1.93% and 2.53% in groups UV and F, respectively (Figure 2d). And the expressions of CD4/ CD8 are 1.53% in group UP (Figure 2e).

### The UVF rat model increased expressions of inflammatory cytokines and reparative factors in the fallopian tubes

**Figure 3:**
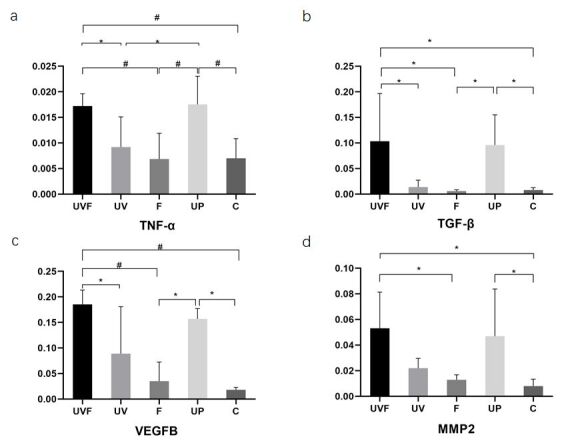
Bar graphs for expressions of inflammatory cytokines and reparative factors in the fallopian tubes and connective tissues. a-d suggest that the expression of TNF-α, TGF-β, VEGFβ, and MMP-2 increased dramatically in the UVF group compared to that in groups F and C (*P<0.05, #*P*<0.01). UP ameliorated TNF-α, TGF-β and VEGFβ expression compared to groups F and C, respectively (*P<0.05, #*P*<0.01). UVF enhanced TNF-α, TGF-β, and VEGFβ expression compared to that in the UV group (*P<0.05). TNF-α expression was lower in the UV group than in the UP group (*P<0.05). The treatments also improved the expression of MMP-2 in the UVF and UP groups compared to that in group C (*P<0.05, #*P*<0.01). MMP-2 levels increased in the UVF group compared to those in group F (*P<0.05), as shown in **Figure 3d.** UVF, vaginal injection of *Ureaplasma urealyticum* liquid combined with fatigue and hunger; UV, rats with vaginal injection of *Ureaplasma urealyticum* liquids; UP, *Ureaplasma urealyticum* liquids were injected into the two uteri during the operation; F, rats under fatigue and hunger; C, control.

Regarding mRNA expression levels of TNF-α, TGF-β, MMP-2, and VEGFβ in the fallopian tubes and connective tissues, as measured by RT-PCR, treatments enhanced their expression in the UVF group. As shown in Figure 3a-c, UVF and UP ameliorated TNF-α, TGF-β, and VEGFβ expression compared to groups F and C, respectively (*P<0.05, #*P*<0.01). UVF enhances TNF-α, TGF-β, and VEGFβ expression compared to that in the UV group (*P<0.05). TNF-α expression was lower in the UV group than in the UP group (*P<0.05). There were no significant differences in the expression of TNF-α, TGF-β, MMP-2, or VEGFβ between the UVF and UP groups (*P*>0.05). In addition, there were no significant differences in the expression of TNF-α, TGF-β, MMP-2, or VEGFβ among the UV, F, and C groups (*P*>0.05). The treatments also improved the expression of MMP-2 in the UVF and UP groups compared to that in group C (*P<0.05, #*P*<0.01). MMP-2 levels increased in the UVF group compared to those in group F (*P<0.05), as shown in Figure 3d.

### The UVF rat model promoted inflammatory cytokines in serum

**Figure 4:**
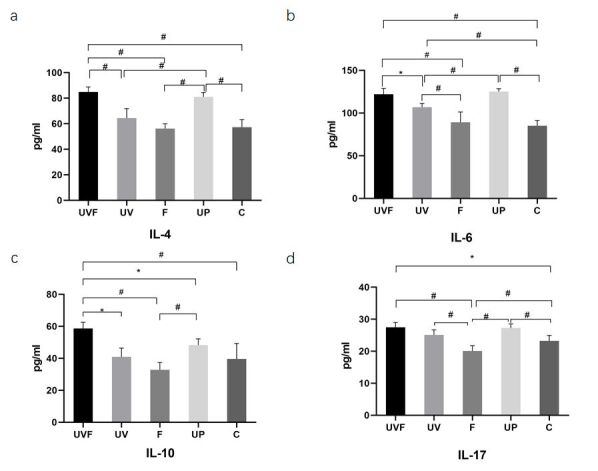
Bar graphs for changes of cytokines in serum using ELISA. a-d show IL-4, IL-6, IL-10, and IL-17 levels in the serum. The expressions of IL-4, IL-6, IL-17, and IL-10 in rat serum in the UVF group increased significantly compared with that in groups F and C by ELISA (*P<0.05, #*P*<0.01). IL-4 and IL-6 levels were elevated in the serum of UP compared to those in the UV and C groups (*P<0.05, #*P*<0.01). The UVF group showed higher levels of IL-4 and IL-6 than the UV group (*P<0.05, #*P*<0.01). UV treatment led to higher levels of IL-6 than in groups F and C (#*P*<0.01). The expression of IL-10 increased in the serum of UVF compared to that in the UV, F, UP, and C groups (*P<0.05, #*P*<0.01), as shown in **Figure 4c.** Augmented levels of IL-4 and IL-10 were found after UP compared with those in group F (#*P*<0.01). IL-17 levels increased in the serum of UVF and UP rats compared to those in groups F and C, respectively (*P<0.05, #*P*<0.01), as shown in **Figure 4d.** Rats that experienced fatigue and hunger showed lower levels of IL-17 than those in the UV and C groups (#*P*<0.01). UVF, vaginal injection of *Ureaplasma urealyticum* liquid combined with fatigue and hunger; UV, rats with vaginal injection of *Ureaplasma urealyticum* liquids; UP, *Ureaplasma urealyticum* liquids were injected into the two uteri during the operation; F, rats under fatigue and hunger; C, control.

The expression of IL-4, IL-6, IL-17, and IL-10 in rat serum in the UVF group increased significantly compared with that in groups F and C by ELISA (*P<0.05, #*P*<0.01), as shown in Figure 4a-d. In addition, there were no significant differences in the expression of IL-4 and IL-10 between the UV, F, and C groups (*P>0.05). IL-4 and IL-6 levels were elevated in the serum of UP compared to those in groups UV and C (*P<0.05, #*P*<0.01), as shown in Figure 4. The UVF group showed higher levels of IL-4 and IL-6 than the UV group (#*P*<0.01). There were no significant differences in expression levels between the UVF and UP groups (*P*>0.05). The UVF group showed higher levels of IL-6 than groups F and C (#*P*<0.01). There were no significant differences in expression levels between the UVF and UP groups *(P*>0.05). The expression of IL-10 increased in the serum of UVF compared to that in the UV, F, UP, and C groups (*P<0.05, #*P*<0.01), as shown in Figure 4c. Augmented levels of IL-4 and IL-10 were found after UP compared to those in group F (#*P*<0.01). IL-17 levels increased in the serum of UVF and UP rats compared to those in groups F and C (*P<0.05, #*P*<0.01), as shown in Figure 4d. Rats that experienced fatigue and hunger showed lower levels of IL-17 than those in the UV and C groups (#*P*<0.01). There were no significant differences in the expression of IL-17 between the UVF and UP groups (*P*>0.05).

## Discussion

Considering the possible damage of fatigue and hunger on the immune system, we introduced a PID rat model by vaginal injection of liquid *Ureaplasma urealyticum* combined with fatigue and hunger. We evaluated the associated inflammatory alterations and expression of proliferation- and repair-related factors to confirm its validity. We found that the rat model led to the generation of some inflammatory cytokines and changes similar to PID alterations. We suspect that the rat model based on vaginal injection of liquid *Ureaplasma urealyticum* combined with fatigue and hunger might be a potential model to further explore the mechanism and therapies for PID.

PID is a common disease of the lower genital tract. The leading cause of lower inflammation is *Ureaplasma urealyticum*. Inflammation influences the upward tracts, such as the uterus, ovaries, and fallopian tubes, and induces more difficult sequelae unless effective and timely treatment is required (Oh et al., 2016). Because the sequelae of PID may be severe, many researchers have focused on PID animal models to explore the underlying mechanisms of PID and potential therapies. The current model was created mainly through the injection of *Ureaplasma urealyticum* after surgical operation. However, surgery and direct injection lead to the death of experimental animals. The study found that tissue damage in PID, such as edema, inflammatory infiltration, and adhesion occurred in the UVF rat model, based on the HE results. In addition, we hypothesize that alterations might be related to the augmented levels of inflammatory cytokines and MMP2 and VEGFΒ associated with vessel production and migration of fibroblasts.

IL-6 and IL-17 are inflammatory cytokines that play a role in immune modulation. IL-6 induces and transmits inflammatory cytokines and promotes the restoration and healing of injured tissue (Tanaka et al., 2014). IL-17 is a proinflammatory factor secreted by active helper T cells (Th17) that improves the production of chemokines such as IL-8 and IL-6, monocytes, and neutrophils. IL-17 enhances local inflammation and damages tissue (Miossec and Kolls, 2012). TNF-α is a proinflammatory cytokine and biomarker of peritoneal connections (Holtmann and Neurath, 2004; Wilson, 2018). IL-4 and IL-10 are anti-inflammatory cytokines that play important roles in immune regulation. IL-4, secreted by Th2 cells, inhibits the function of pro-inflammatory cytokines and plays a vital role in the negative feedback of inflammation (Mitchell et al., 2017). IL-4 also prevents the excessive activity of inflammatory cells (Monteiro et al., 2018). IL-10 reduces the generation of pro-inflammatory cytokines and inflammation (Taylor et al., 2009). TNF-α has been reported to increase expression in the serum and irrigation solution of peritoneal connective tissue in rats (Jaafari et al., 2021). TGF-β is a multifunctional protein that often increases inflammation. Using ELISA or RT-PCR, we found that the UVF rat model dramatically enhanced the expression of IL-6, IL-17, IL-4, IL-10, TNF-α, and TGF-β. These results support the idea that model rats possess inflammatory mediators involved in PID. Moreover, some typical inflammatory appearances, such as edema of the fallopian tubes and connective tissues, might be caused by higher levels of inflammatory cytokines, such as IL-6, IL-17, IL-4, and IL-10. Additionally, this model might enhance the production of inflammatory mediators via abnormal activation of TNF-α and TGF-β. These results confirmed our assumption that these rats could be a potential model for PID-associated research.

MMP-2 degrades collagen IV and fibronectin and promotes the production and migration of fibroblasts. MMP-2 can be induced by many inflammatory cytokines and plays an essential role in the destruction of basement membrane integrity, restoration of injury, and scar formation (Alizadeh et al., 2017; Saad et al., 2002). VEGF is a cytokine that improves vessel production. VEGF increases capillary permeability and enhances inflammation by directly binding to VGEF receptors. Inhibition of VEGF expression can decrease capillary formation and prevent tissue injury and adhesion (Molema et al., 2022). In our study, the UVF rat model showed significantly increased MMP-2 and VEGF expression. The infiltration of inflammatory cells and adhesion are likely related to the upregulation of MMP-2 and VEGF, which play essential roles in migration, invasion, and vessel production.

The T lymphocyte subpopulation mainly controls cellular immunity, functions as resistance to viruses, and modulates immunity. It depends on the proportion of T lymphocytes (CD3+) and the ratio between lymphocyte subpopulations (CD4+ and CD8+). The equilibrium between subpopulations is maintained under average conditions; however, diseases occur when the balance and proportion are disturbed. CD3 is expressed on all surfaces of T lymphocytes and is representative of cellular immunity. CD4+ is the ratio of inducible T lymphocytes (Ti) to helper T lymphocytes (Th), and is recognized as a significant factor in immunity. CD4+ regulates the pivotal cells in cellular immunity (Brown et al., 2019). CD8+ is the ratio between suppressive T lymphocytes (Ts) and cytotoxic T lymphocytes (Tc), and inhibits immunity (Ilatovskaya et al., 2019). The differences after *Ureaplasma urealyticum* liquid injection suggest that *Ureaplasma urealyticum* causes immune disorders. The differences in results between rats under fatigue and hunger and control rats suggest that fatigue and hunger could damage the immune balance.

## Conclusion

We suggest that a rat model of vaginal injection of *Ureaplasma urealyticum* liquid, combined with hunger and fatigue, can cause inflammatory alterations such as adhesion, obstruction, and edema. It also leads to the production of inflammatory cytokines, immune disorders, and invasion-associated genes. Moreover, inflammatory alterations reinforce tissue damage. In conclusion, the established rat model could mimic some specific inflammatory impairments of PID and be applied in future research to explore its mechanism and potential therapies.
